# Decreased cortisol among hikers who preferentially visit and value biodiverse riparian zones

**DOI:** 10.1038/s41598-020-79822-w

**Published:** 2021-01-13

**Authors:** Ellie Opdahl, Kathryn Demps, Julie A. Heath

**Affiliations:** 1 Department of Biological Sciences, Boise State University, Boise, ID 83725 USA; 2 Department of Anthropology, Boise State University, Boise, ID 83725 USA

**Keywords:** Ecosystem services, Riparian ecology, Biomarkers, Conservation biology, Environmental impact, Psychology and behaviour

## Abstract

While outdoor recreationists often report increases to their well-being for time spent in nature, the mechanisms through which local ecologies affect human health have been difficult to quantify, and thus to manage. We combine data from pre-post salivary cortisol measures, GPS tracks, visitor photos, and surveys from 88 hikers traversing several types of landscape within peri-urban public lands in southwest Idaho, USA. We find that time in biodiverse riparian areas and areas of perceived aesthetic value correlates with decreases in salivary cortisol and improved well-being for hikers. Wildlife sightings were not associated with changes in salivary cortisol, but were associated with riparian travel and aesthetic preferences, indicating an indirect pathway for ecosystem services. Additionally, wildlife sightings decreased on high-use days, even though hikers did not perceive a negative impact of their recreational activity. These results suggest that cultural and physiological ecosystem services of nature depend on the ecological community of the area. Preferential visitation and high service value of riparian areas by hikers and wildlife alike target shared riparian areas as hot spots for management efforts to promote both ecological and human health within an increasingly urbanizing world.

## Introduction

Outdoor recreational areas highlight the importance of cultural ecosystem services that are significant, yet underrepresented contributors to human well-being^1,2^. Spending time in nature is associated with physical, psychological and physiological benefits for human well-being^3–7^. Though humans report responding positively and restoratively to nature, the quantitative mechanisms behind this relationship remain unknown. Some have hypothesized that humans have an innate tendency to affiliate with nature, which developed over evolutionary time from a biophilic contribution to well-being^8^. While exposure to biodiversity is implicated in this research, the mechanisms by which it is perceived and translated into well-being needs further investigation.

Previous research suggests that the landscape itself may be an important cue for human well-being. Water is associated with positive impacts to human perceptions of aesthetics, increased biodiversity, and increased urban quality of life^9^. Water bodies, or “blue spaces,” are associated with reported increases in mood and self-esteem, as well as decreases in psychological stress^3^. Forested landscapes and higher amounts of “greenness” may be associated with greater psychological and physiological benefits as well^10,11^. However, little work has been done to associate the biodiversity and wildlife of a natural landscape to human well-being^12^. By connecting human well-being benefits to landscape ecology, we hope to bring novel insights into the mechanisms and relationships behind nature restorative effects, as well as pathways for preserving the many services provided by outdoor recreational areas.

Countermanding their societal benefits, outdoor recreation activities are associated with negative environmental consequences such as the degradation of habitat and disturbance to wildlife^13^. Yet, participation in outdoor recreation continues to grow. In 2016, the Outdoor Industry Association estimated that almost half (48.8%) of the United States population participated in outdoor recreation at least once. Land managers are challenged to maintain the social carrying capacity of a recreational area with little quantitative data on how the patterns and effects of recreation degrade natural resources. This is as crucial for sustaining healthy ecosystems as it is for providing positive visitor experiences^9^. Meanwhile, there has been little research investigating visitor use within the context of cultural ecosystem services and responses of human well-being. Understanding the human–environment interactions between recreationists and nature is imperative for continued sustainable use and provision of ecosystem services. Additionally, if the increase in well-being is strongest in urban dwellers and individuals experiencing heightened stress-levels^14^, peri-urban natural areas may be particularly salient venues to maintain recreational ecosystem services.

We investigate these interactions in a peri-urban recreational area on public lands in the Boise Foothills located in southwest Idaho, USA. Characterized with both increasing visitor use and variable landscapes, the Boise Foothills are easily accessed by an urban population attempting to relax and rejuvenate in a variety of natural and social settings. We combine social and biological science in a mixed methods approach to identify the mechanisms through which recreationists perceive and benefit from the environment. Using a pre-post paired design, we sampled hikers at a prominent trailhead for salivary cortisol before and after their hiking trip. Hikers were defined as individuals who were intent on walking in the recreational area, and excluded runners, bikers, and other more exertive recreational users. Hikers carried a handheld GPS device with them to track their route, and were asked to take photographs of landscapes they found particularly beautiful. At the end of their trip, hikers completed a survey about aesthetic preferences, perceived ecological impact and knowledge of the landscape, recreational motivation, and total wildlife observations. The difference in hiker salivary cortisol concentration was then connected to GPS tracks, photos, and survey data to link cortisol change to experiences of the local ecology such as land cover type, perceived aesthetic quality, and observations of wildlife. We also tied hiker activity back to land management and evaluated perceived recreational impact, wildlife observance, and visitation rates within the greater context of conservation.

## Results

**Figure 1:**
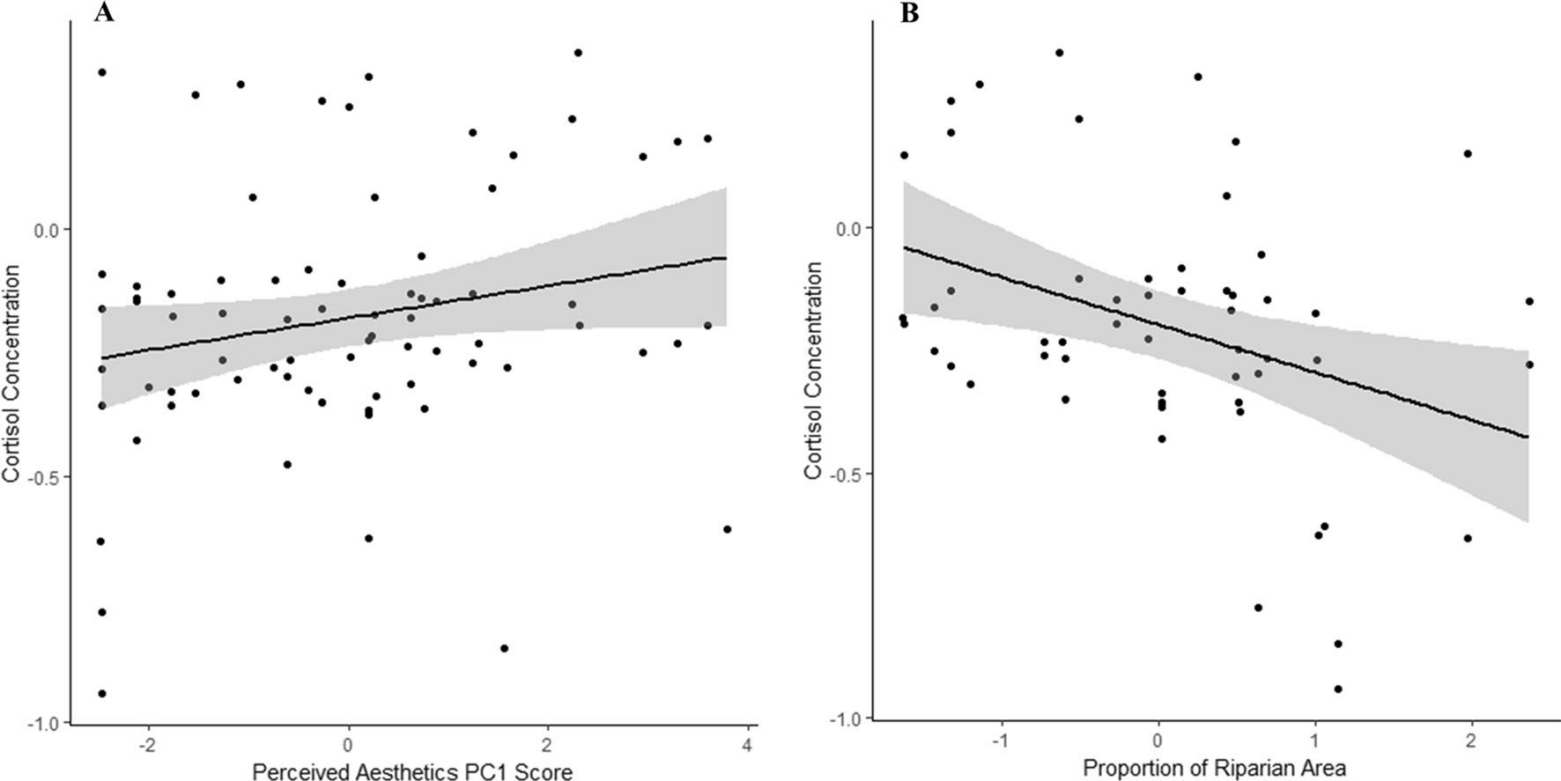
The relationship between the change in cortisol (µg/dL) and both perceived aesthetics and riparian area with best fit lines and standard error (n = 55). (**A**) Cortisol significantly increased after recreating in low aesthetically perceived landscapes. (**B**) Cortisol significantly decreased after recreating in more riparian areas. Both figures are plotted against the residual change in cortisol when controlling for either aesthetic PC1 score or riparian area.

After taking into account the effects of cortisol’s diurnal cycle and hiker demographics, we found that cortisol decreased in hikers who recreated through more riparian areas (*n* = 55, *F*_*1,52*_ = 8.575, *P* < 0.01) (Fig. 1A), and who perceived the aesthetic quality of the surrounding landscape to be high (*n* = *55. F*_*1,52*_ = 6.069, *P* < 0.05). Riparian areas were also associated with high perceived aesthetic quality (Fig. 1B). All photo points (*n* = 22) of high aesthetic areas taken by hikers were clustered in areas with high vegetation (median KDE: 13.381), water (40.535) and riparian (23.981) cover. Photographs of highly aesthetic areas also commonly depicted water, mixed vegetation, and wildlife.

Though wildlife observations did not impact the change in hiker cortisol concentrations (*n* = 55, *F*_*1,49*_ = 2.190, *P* = 0.145), wildlife may indirectly affect human well-being through their contribution to ecological diversity. Wildlife were often portrayed in photographs of high aesthetic riparian areas, and were observed at significantly higher numbers in riparian zones compared to all other land cover classes (*n* = 47, *Χ *^*2*^ = 4.618, *P* < 0.05). We found that on weekends when visitor use was higher, wildlife observances by hikers significantly dropped compared to less visited weekdays (*n* = 47, *Χ *^*2*^ = 6.033, *P* < 0.05). Despite the effect of visitor use on wildlife presence, hikers who were more motivated to view wildlife perceived their impact on wildlife to be positive rather than negative (*n* = 59, *r*_*s*_ = − 0.290, *P* < 0.05).

## Discussion

**Figure 2:**
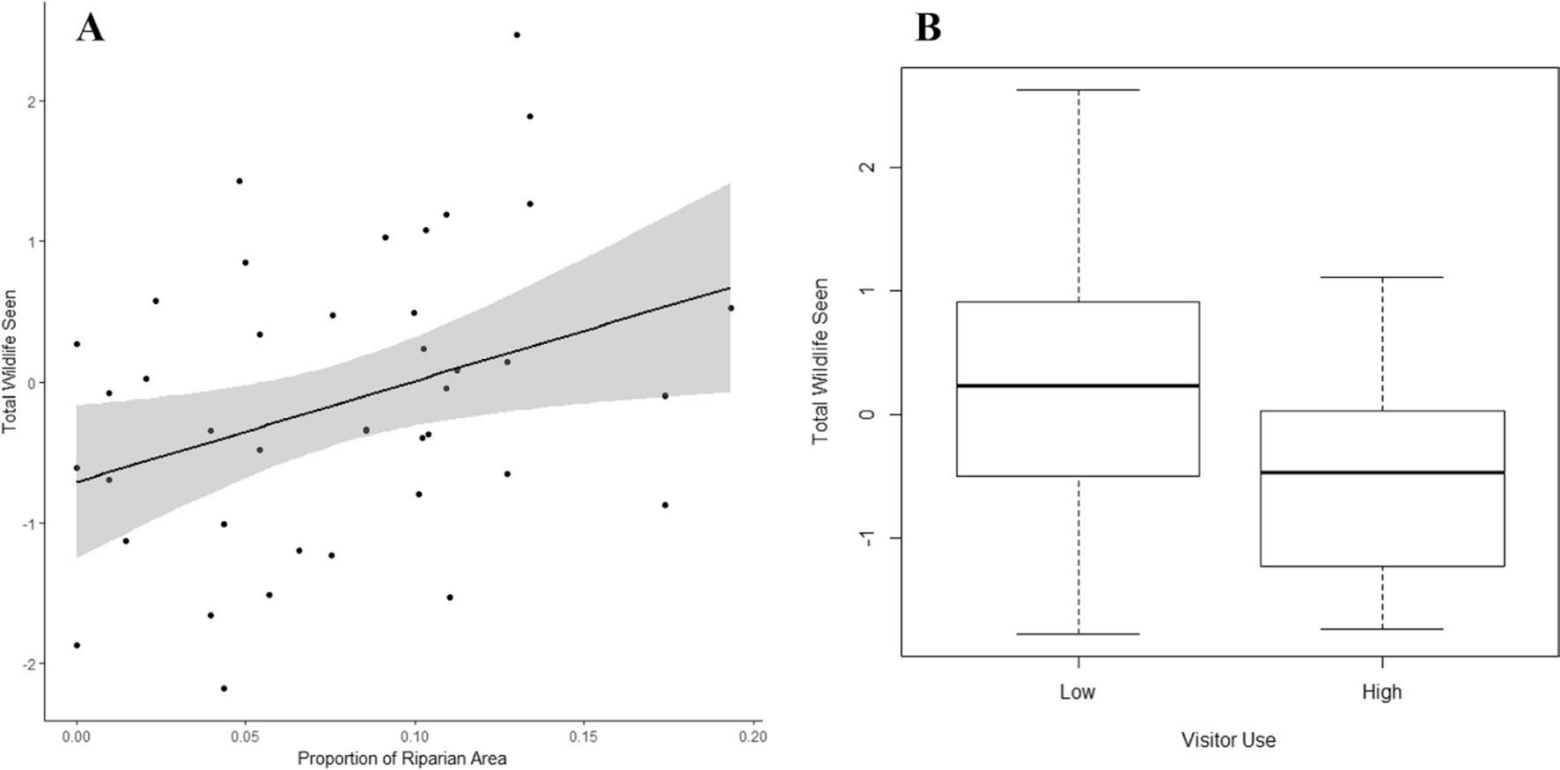
The relationships between the number of wildlife seen while recreating in riparian areas and by high/low visitor use (n = 47). (**A**) Wildlife observances significantly increased with increasing riparian area. (**B**) Wildlife observances significantly increased on low visitor use days compared to high visitor use. Both figures are plotted using the residual change in total wildlife seen when controlling for riparian area, as well as recreational crowding.

**Figure 3:**
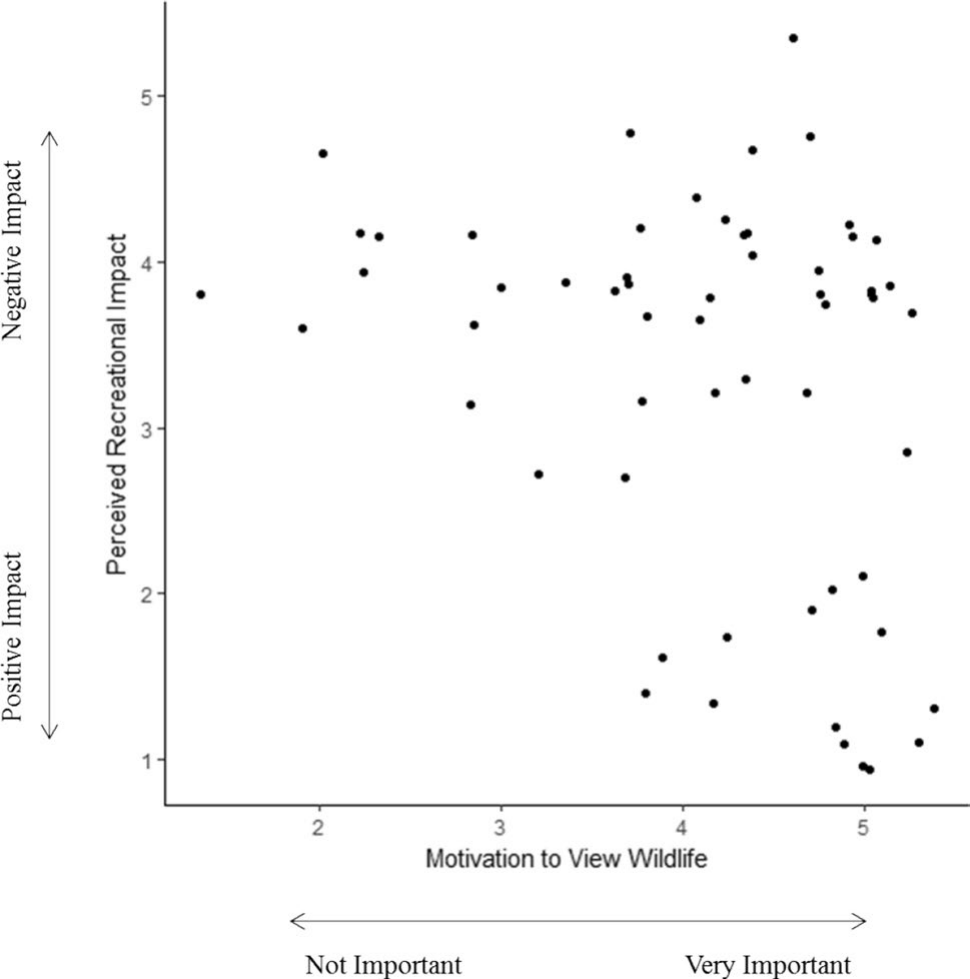
Ordinal rankings of perceived recreational impact to wildlife (1 = Positive, 3 = No impact, 5 = Negative) compared to motivation to view wildlife (1 = Not Important, 3 = Neutral, 5 = Very important) (n = 59). There is a negative correlation between viewing wildlife and perceived recreational impacts.

Our results use an interdisciplinary human–environment systems approach to elucidate hitherto underexplored connections between outdoor recreationists, well-being, and the landscape. Incorporated into blue and green spaces, we found that biodiversity may provide a pathway to human well-being. Hikers’ salivary cortisol decreased, and decreased more, as they passed through more biodiverse riparian areas and landscapes they perceived to be aesthetically pleasing (Fig. 1). Though wildlife presence was not associated with decreases in cortisol, wildlife did contribute to aesthetic perception and were considered important components to beautiful landscapes. Hikers who value and benefit from these landscapes, though, underestimate their impact on biodiversity, and thus future use value. Fewer wildlife sightings occurred on busy weekends (Fig. 2), though many recreationists did not perceive any negative impacts to wildlife (Fig. 3). The preservation of biodiversity, therefore, could better connect conservation goals with society by helping to protect both ecological integrity and resilience, as well as sociological and cultural services.

**Figure 4:**
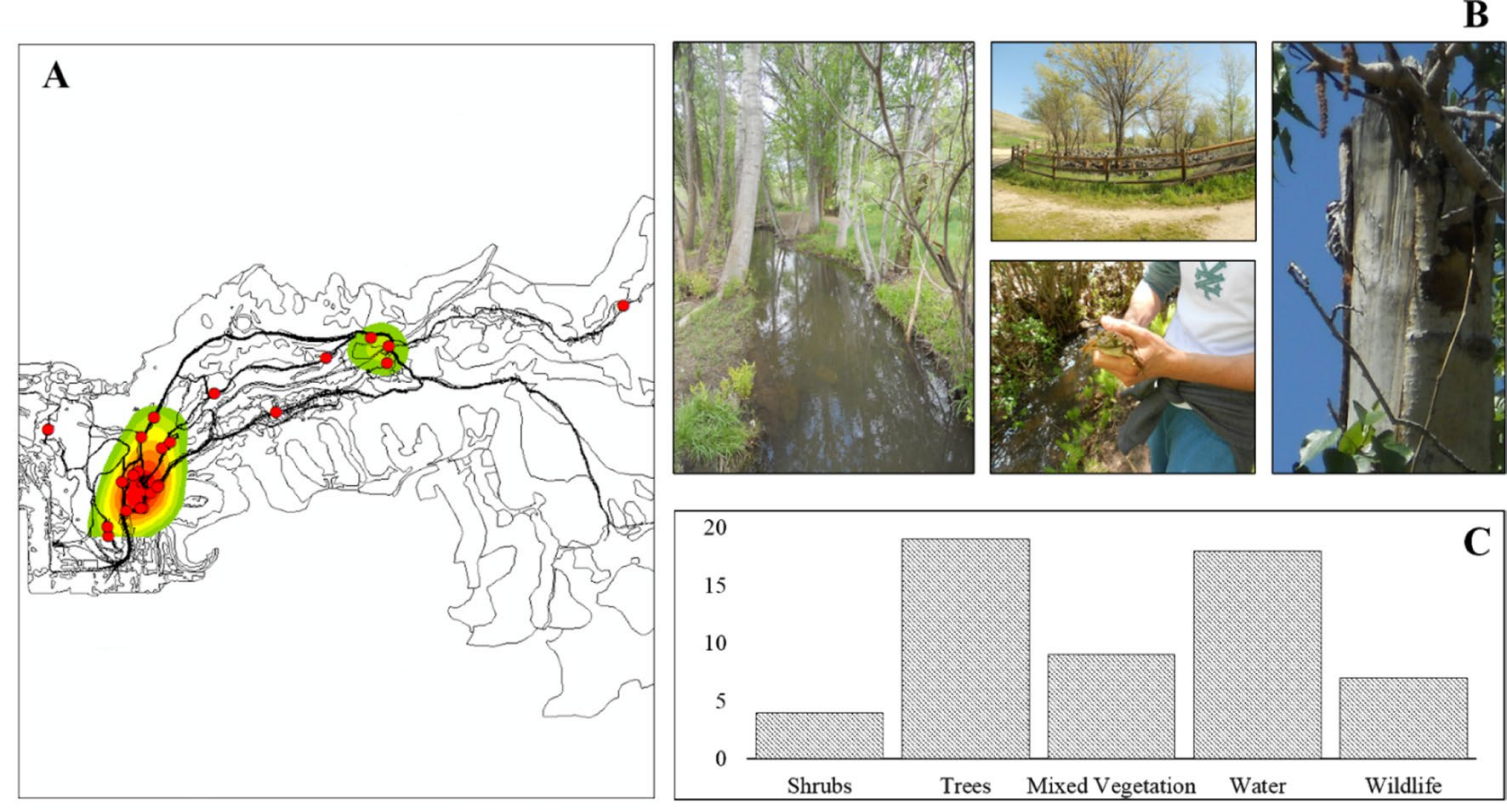
Volunteer employed photography results including (**A**) kernel density estimate (KDE) of photo points, (**B**) a sample of representative photographs from the area of highest photographic density, and (**C**) a bar graph depicting the various photographic elements captured by each participant (n = 22). KDE estimates are shown alongside land cover type boundaries, participant tracks (black), and photo points (red). Densities are depicted from highest to lowest where red refers to high photographic density, and green as low photographic density.

Hikers preferentially utilized trails traversing riparian areas, and hikers that had greater reductions in salivary cortisol were more likely to have passed through riparian land cover when recreating. These patterns follow similar indicators of the value of outdoor blue and green space in previous studies^12,15^. Hikers in this context considered riparian areas to be more beautiful, with high aesthetic quality being attributed to the presence of water, trees, high vegetation, and wildlife (Fig. 4).

It is interesting to note that though wildlife sightings did not correlate with changes in cortisol, wildlife may have an indirect effect by contributing to the overall aestheticism of the landscape. Previous studies characterize riparian areas as highly biodiverse^3^. We found that hikers were more likely to report seeing wildlife while passing through riparian areas over any other land cover type. Biodiversity can be a sign of ecological health and integrity, and could be a good indicator for areas with high quality and quantity of ecosystem services. It is possible that recreationists receive more well-being benefits when surrounded by high quality habitat that feels natural, healthy, and ecologically complex.

Of course there are many limitations to this study and thus future directions for research indicated by these results. Others have found within-individual effects of walking in nature versus urban areas, with greater decreases in salivary cortisol following exposure to natural areas^16,17^ indicating that self-selection for outdoor recreation is unlikely to be driving the observed effects. Urban dwellers prescribed a ‘nature pill’ also showed decreases in salivary cortisol following exposures to natural areas^18^. A meta-analysis of 22 studies in Asia and Europe shows our results to be robust to walking in forested landscapes^19^– we replicate this effect in a natural area where people can choose between forested and non-forested landcover. Although the direction of the effect remains the same, more research is needed on the role of cultural and personal perceptions and experiences of natural areas and the impact on well-being. Beyond visual cues, the sounds and scents of a forest have been implicated for increasing human well-being during nature walks^19^. However, when hikers in our study traversed the same areas, individuals who perceived the habitat as degraded and with low aesthetic value had increased cortisol profiles compared to those who perceived it as un-degraded with high aesthetic value (Fig. 1). There may be some self-selection for aesthetic preference, but the mechanisms for translating nature exposure to improved well-being seem to be at least partially derived from personal experience.

In addition to their preferential views and habitat type, the riparian trails of the Boise Foothills are used as connector trails to other parts of the ridge-to-rivers trail system, and are thus the most susceptible to overuse. High visitor use could lead to degraded environments, fewer ecosystem services, and a general reduction of visitor experience^9,15^. In fact, fewer wildlife sightings were reported during weekends when visitor use was higher. This suggests that wildlife in the region may be subject to future displacement relating to high recreation in riparian areas as use continues to increase. The high valuation of riparian areas for both humans and wildlife makes riparian zones a hotspot for assessing possible conflicts between the human and natural systems. If wildlife indirectly affects landscape aesthetic quality and human well-being, any changes in wildlife stress, behavior, or distribution could then reduce both environmental and experiential quality^20^.

However, threats to natural areas do not seem to be a concern for individual hiker behavior. Despite scientific research that suggests outdoor recreation negatively affect wildlife, most people in our study did not feel that their hiking trip had any negative impact on wildlife: 63% perceived recreation as having a positive impact, 12% as having no effect, and only 25% of hikers perceived recreation as having a negative impact on wildlife. Of those who perceived recreation as having a positive impact on wildlife, the group was dominated by people whose primary motivation to engage in outdoor recreation was to observe wildlife. Regardless of motivation, many recreational groups view their own recreational activity as benign, choosing to blame other recreational activities for disturbances to wildlife^13,21^. The perceived positive impact by wildlife viewers could also be due to a disconnect between individual values and behavior, or else a belief that valuing wildlife results in greater success of conservation and management strategies^22^. If recreation continues to be popular in biodiverse habitats where wildlife viewing is higher, future management should endeavor to curb hiker behavior by encouraging wildlife viewing in predictable areas such as by discouraging off-trail use, creating wildlife viewing platforms, and posting educational signs in high traffic areas.

Riparian areas offer a multitude of ecosystem services such as wildlife habitat, wildlife viewing, and ecosystem service benefits from outdoor recreation. The high prevalence of both wildlife sightings and visitor use in riparian areas suggest that riparian zones may be at higher risk for wildlife disturbance and displacement, as well as environmental degradation. To preserve the functionality of riparian zones for both the human and natural systems, visitor spatial use and wildlife populations should be periodically monitored^13,21–24^. Riparian restoration efforts and strategic educational signs may also be necessary to maintain the serviceability of riparian areas and promote more mindful recreation habits. The addition of more trails in riparian areas may also reduce the effect of visitor use by redistributing visitor flow pathways across a broader area. Introducing more trails would allow greater access to benefits and services afforded by riparian zones, as well as reduce off-trail usage and associated environmental degradation. In some cases, wildlife can habituate to recreational use along predictable trail routes^13^. Habituation, however, varies with species, and a biological review of the effect of recreational activity on wildlife should be done prior to establishing new trails.

By using a human–environment systems approach to evaluating human well-being benefits from outdoor recreation, we can build a more comprehensive understanding of how humans and landscapes interact. The analysis of quantifiable cultural ecosystem services is possible, and should be used for assessing the ecological and sociological value of outdoor recreational areas in the future. In shared green spaces, it is possible for wildlife conservation and society to find common ground.

## Methods

### Study areas

We collected survey and salivary hormone data from people at the Camel’s Back—Hulls Gulch Reserve of the Ridge to Rivers Management Area in Boise, Idaho, USA (Fig. 1S). The Lower Hulls Gulch area is largely characterized by sagebrush steppe habitat as well as riparian areas that act as habitat to a variety of avian, mammal, and herpetological species. Common wildlife spotted by recreationists include birds, amphibians, reptiles, and small mammals. We collected data between March 24—May 18, 2017 on both the weekend and weekdays. Weather data, consisting of average wind speed and average air temperature were taken from the Crestline Trail Idaho (Boise, ID).

### Participants

We recruited participants at a prominent trail head associated with the recreational area, and the purpose and protocols of the study were explained. Upon agreeing to participate, participants were asked to read an additional written statement of the project and sign a informed consent form. Each recruited individual was minimally required to participate in either the saliva collection or survey, although participation in all parts of the study was highly encouraged. Any recreationists who had been recreating for more than 10 min were not recruited. We defined hikers as recreationists who were intent on walking in the recreational area, and excluded users who were performing exertive activities such as running or biking. All participants were given an anonymous identifier which was used throughout the study. This research project was reviewed and approved by the IRB at Boise State University, Idaho, USA under #006-SB17-061.

### Salivary cortisol and testosterone collection

We restricted all sampling until late morning (often 10:00am) to control for diel patterns in hormone concentrations after awakening^25–27^. To further account for any variations due to time of day, the time of collection was recorded for each saliva sample and factored into each analysis. We found that the time of day each cortisol sample was taken did not account for variation, and that the diurnal cycle of cortisol did not explain the change in cortisol concentration that we observed in hikers.

Saliva samples were collected from recreationists using a pre-post paired design. Participants were asked to give at least 0.25 mL of saliva using a saliva collection aid (2 mL cyrovial, Salimetrics PA, USA) via the passive drool method^25,28^. The passive drool method allows for the collection of large samples for multiple assays, reduces the risk of contamination by collection substances, and allows samples to be frozen without interfering with assay protocols^28^. Saliva was immediately stored in a portable cooler with ice until frozen at -10 °C. Cortisol and testosterone concentrations were assessed using a Salimetrics Cortisol Enzyme Immunoassay following the manufacturer’s protocols and design (Salimetrics, PA, USA). All assay plates were read using the Gen5 software and Biotek EL800 Plate Reader. Hormone concentrations were then calculated from the optical densities using a standard curve and the online elisaanalysis interface.

### Survey collection

After recreating, all participants were asked to take a survey. Survey questions included the following: variables affecting psychological stress levels, observation of wildlife, motivations for recreation (ranging from social, personal challenge, wildlife, and solitude), perceived ecological impact that outdoor recreational activity has on wildlife and habitats, and basic demographics including age and gender. In addition, participants were asked to rate how psychologically stressed they felt after their recreational activity. Participants also had the opportunity to note any negative experiences they had while recreating.

### GPS track collection

Participants were encouraged to carry a handheld GPS device to track their route while hiking. We used GPS tracks to connect hiker routes with land cover types such as % vegetation cover, urban cover, water, and riparian cover. Using a 100 m buffer as a standard measurement for visibility, we calculated the proportion of land cover types each hiker traveled through during their trip. We also used GPS tracks to compare line density calculations to estimate areas and trails with higher recreational traffic.

### Photograph collection

We used volunteer employed photography and asked participants to take photographs of landscapes they found beautiful. At the end of the trip, participants were asked to select one photograph that captured an area of high aesthetic value to them. Photographs were spatially linked to the landscape using participant GPS tracks, and analyzed using kernel density estimates (KDE) to compare what areas and land cover types were photographed more frequently. In addition, the subject and photographic elements of each photograph were tallied and collected to compare what components of the landscape participants were more likely to take pictures of.

### Statistical analyses

To assess the change in salivary cortisol to landscape aesthetics, we used a backward stepwise approach to create a linear model with the following predictor variables: perceived aesthetics (using principal component PC1 scores), land cover metrics, start time, duration, total wildlife observance, plant identification skills (as a measure of ecological familiarity), and all aesthetic interaction terms. . We checked for collinearity among variables and considered pairwise r >|0.70| to indicate a correlation. Due to high correlation between start time and duration, duration was removed from all models. A total of 6 saliva samples (all hikers) were not used due to unusually high (> 2 SD) cortisol concentrations.

Non-significant interaction variables were removed first based on lowest SS values, followed by main effect variables with the lowest SS value until only statistically significant variables remained. Cortisol was evaluated as the change in concentration from recreational activity (Post-collection—Pre-collection). Negative changes correspond to a decrease and positive changes to an increase in hormone concentration after recreating. To meet normality assumptions, the change in both cortisol was transformed using a square root function taken at absolute value. After data transformation, all original signs were returned to maintain the negative–positive spectrum of hormone change. The removal of any outliers did not change the interpretation of the results and were kept across analyses.

We used a backward stepwise approach to explain aesthetic perception with the following predictor variables: weekend/weekday (as a metric of visitor use), total wildlife observance, plant identification skills (as a metric of ecological familiarity), gender, age, duration, and all interaction terms. We used a backward stepwise approach and a generalized linear model to evaluate whether land cover, recreational use, and ecological familiarity explained the number of wildlife observations.No variables were correlated with each other, and all interaction terms were included. To investigate the relationship between perceived ecological impact metrics and recreational motivation, we used spearman correlations.

## Supplementary information

Supplementary Information 1.

## Data Availability

The raw dataset is available through the Boise State library database under 10.18122/MILES/26/boisestate
