# Supplementary material for: Decreased cortisol among hikers who preferentially visit and value biodiverse riparian zones
Source: Sci Rep. 2021 Jan 13;11:848. doi: 10.1038/s41598-020-79822-w (PMC7806922; doi:10.1038/s41598-020-79822-w)
Supplement: Supplementary file 1 — Supplementary Information 1. [file 41598_2020_79822_MOESM1_ESM.pdf]

Decreased cortisol among hikers who preferentially visit and value biodiverse riparian zones

Authors: Ellie Opdahl[1, 2], Kathryn Demps[2*], Julie A. Heath[1]
Affiliations:
[1]Department of Biological Sciences, Boise State University, Boise, ID 83725, USA
[2]Department of Anthropology, Boise State University, Boise, ID 83725, USA
*Correspondence to: kathryndemps@boisestate.edu

Please answer the following questions on recreational activity.

	Hiking/Walking	Mountain Biking	Off-Highway Vehicle (OHV) Riding
Please select the recreational activity you did today.			
In the last 30 days, how many times have you done each of the three recreational activities listed here?			

Thinking about your recreational experience today…

How many people (other than yourself) did you recreate with today? ___________

Which trails did you use today? (A trail map of the area is available upon request if needed – just ask!)

____

Please list any wildlife you saw while out recreating below.

____

How many animal species (including birds, mammals, and reptiles) did you recognize while out recreating? ___________

How many plant species did you recognize while out recreating? ___________

How comfortable do you feel at identifying wildlife? 1 2 3 4 5 6
Not comfortable — Very comfortable

How comfortable do you feel at identifying plants? 1 2 3 4 5 6
Not comfortable — Very comfortable

How well do you know this recreational area and its trail system? 1 2 3 4 5 6
Not well — Very well

How beautiful did you find the landscape in which you recreated in today? 1 2 3 4 5 6
Not beautiful — Very beautiful

How peaceful did you find the landscape in which you recreated in today? 1 2 3 4 5 6
Not peaceful — Very peaceful

How scenic did you find the landscape in which you recreated in today? 1 2 3 4 5 6
Not scenic — Very scenic

How natural/wild did you find the landscape in which you recreated in today? 1 2 3 4 5 6
Not natural/wild — Very natural/wild

What was your most positive experience during your recreational activity?

____

What was your most negative experience during your recreational activity?

____

How do you feel after your recreational experience today? 1 2 3 4 5
Not stressed — No change — Very stressed

	1	2	3	4	5
	Not stressed		No change		Very stressed

How important were the following things for you to experience during today's activity?

	Not important	Not very important	Neutral	Somewhat important	Very important
Meet new people who enjoy similar things					
Enjoy the natural environment					
Challenge yourself / develop skills					
Enjoy solitude / "get away from it all"					
View and appreciate wildlife					
Share time with friends / family					

How do you think recreationists and recreational activity affect wildlife?

1	2	3	4	5
Positively		No effect		Negatively

Please order the following recreational activities (from 1-5) by how much you think they impact wildlife. 1 is least impactful and 5 is most impactful to wildlife.

Hiking/Walking _____
Mountain Biking _____
Off-Highway Vehicle Riding _____
Birding _____
Running _____

How important is it for the Ridge to Rivers area to provide the following?

	Not important	Not very important	Neutral	Somewhat important	Very important
Place for recreational activities					
Opportunities to know and contact nature					
Cultural significance / sense of place and identity					
Educational opportunity and significance					
Aesthetic/ scenic enhancement of surrounding area					
Wildlife habitat					
Species conservation					

For each pair of photographs, please circle which landscape you would prefer to see while out recreating.

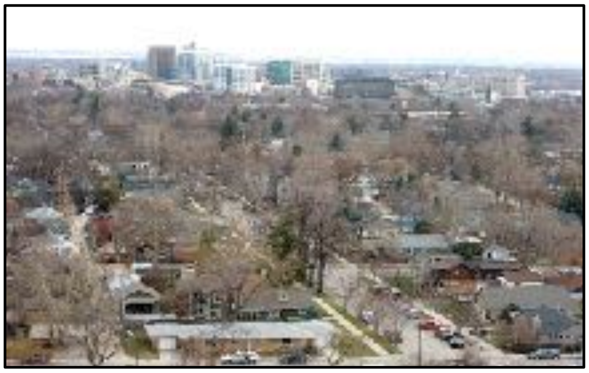

A

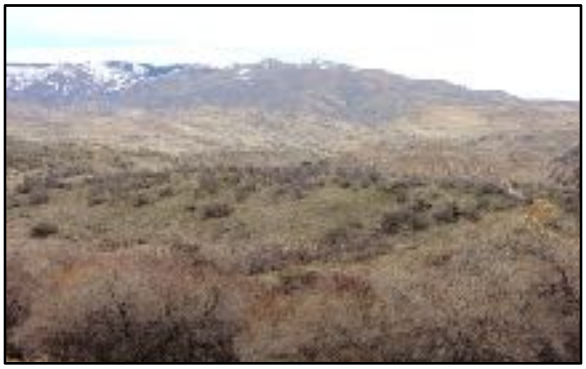

B

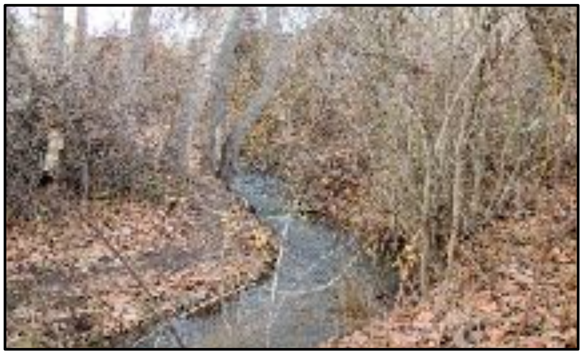

A

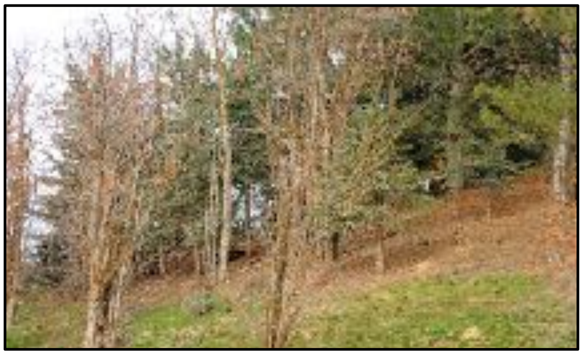

B

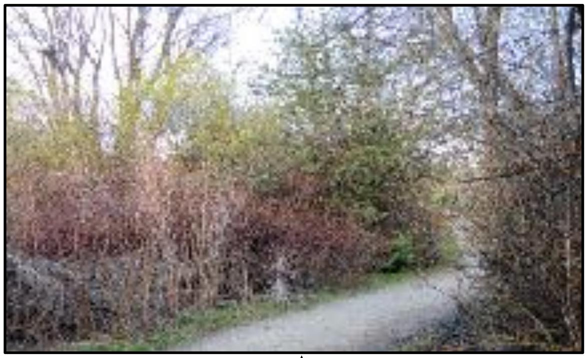

A

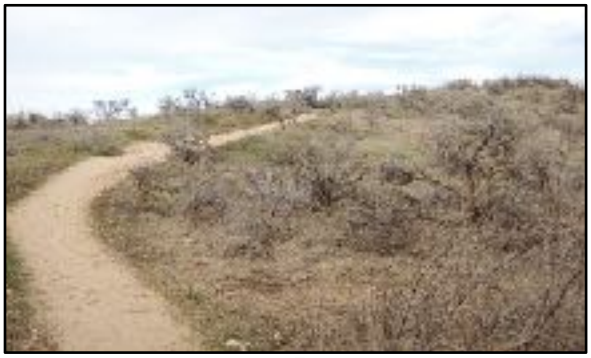

B

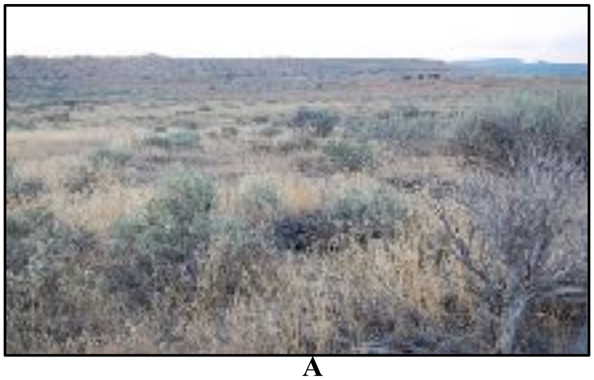

A

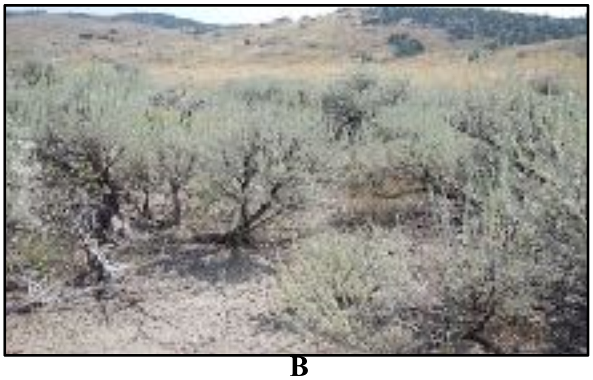

B

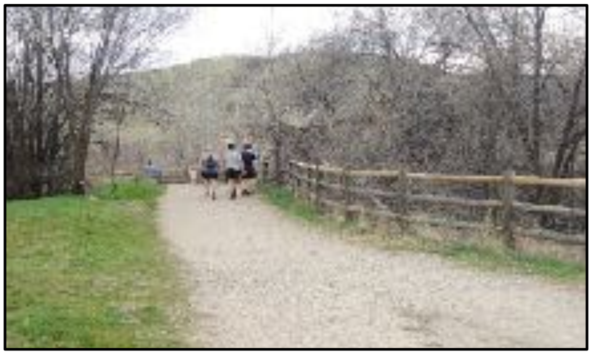

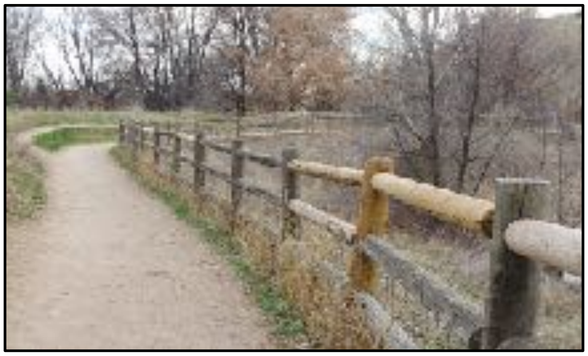

For the following characteristics, please write the letter of the picture you think is the best match.

Most scenic	_________	Most inspiring	_________
Most peaceful	_________	Most wilderness	_________
Most beautiful	_________	Most calming	_________
Most comfortable	_________	Most natural	_________

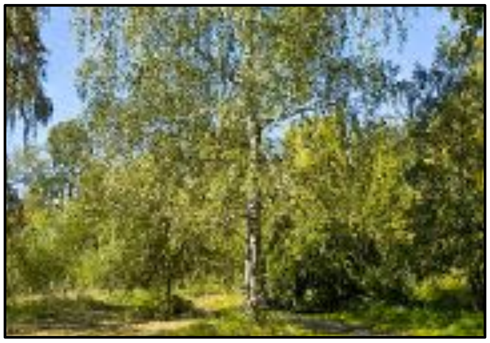

A

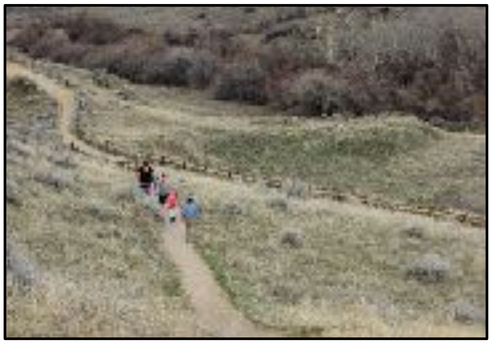

B

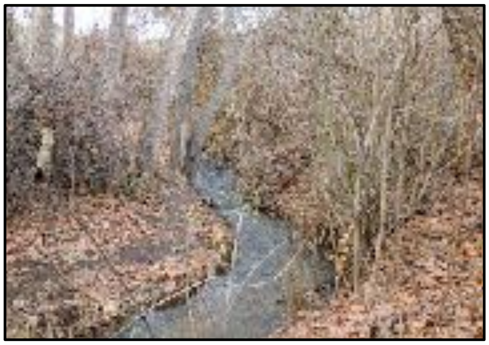

C

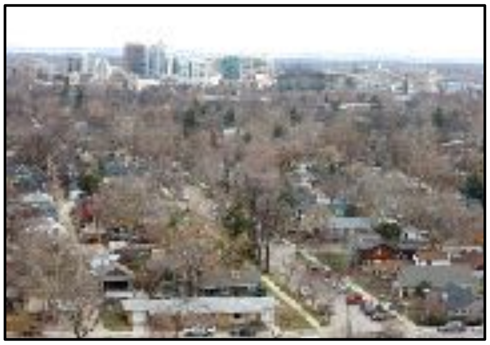

D

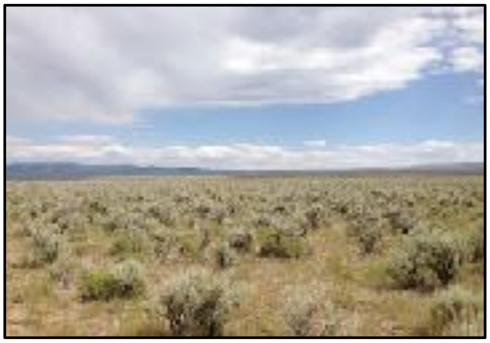

E

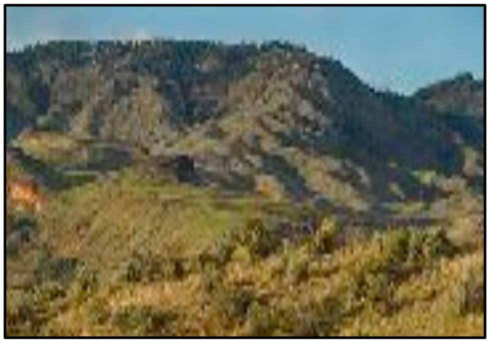

F

Finally, please answer some questions about you…

What type of landscape did you grow up in? Please circle your answer(s).

Desert
Mountains
Shrublands
Forest (please specify what type):_____________________

Grasslands
Agricultural farm or range land
City/Urban
Other: _____________________

How many years have you lived in Boise? ________________

Age:__________

Please circle which gender you identify as: Female Male

Thank you for your time and have a great day!
